# When is Sessional Monitoring More Likely in Child and Adolescent Mental Health Services?

**DOI:** 10.1007/s10488-016-0725-6

**Published:** 2016-02-19

**Authors:** J H. Edbrooke-Childs, D. Gondek, J. Deighton, P. Fonagy, M. Wolpert

**Affiliations:** Evidence Based Practice Unit, UCL and the Anna Freud Centre, London, UK; Anna Freud Centre, 12 Maresfield Gardens, London, NW3 5SU UK; Research Department of Clinical, Educational and Health Psychology, UCL, Gower Street, London, WC1E 6BT UK

**Keywords:** Sessional monitoring, CAMHS, Child, Adolescent, Case complexity

## Abstract

Sessional monitoring of patient progress or experience of therapy is an evidence-based intervention recommended by healthcare systems internationally. It is being rolled out across child and adolescent mental health services (CAMHS) in England to inform clinical practice and service evaluation. We explored whether patient demographic and case characteristics were associated with the likelihood of using sessional monitoring. Multilevel regressions were conducted on *N* = 2609 youths from a routinely collected dataset from 10 CAMHS. Girls (odds ratio, OR 1.26), older youths (OR 1.10), White youths (OR 1.35), and youths presenting with mood (OR 1.46) or anxiety problems (OR 1.59) were more likely to have sessional monitoring. In contrast, youths under state care (OR 0.20) or in need of social service input (OR 0.39) were less likely to have sessional monitoring. Findings of the present research may suggest that sessional monitoring is more likely with common problems such as mood and anxiety problems but less likely with more complex cases, such as those involving youths under state care or those in need of social service input.

Sessional monitoring of treatment progress during psychological therapy involves the regular review of feedback from measures of symptoms, functioning, or common factors such as therapeutic alliance reported by patients or therapists (Carlier et al. 2012). It is an evidence-based intervention recommended by healthcare systems internationally (SAMSHA’s National Registry of Evidence-Based Programs and Practices 2015). Sessional monitoring is being rolled out across child and adolescent mental health services (CAMHS) in England as a means of supporting clinical practice and to underpin evaluation of service provision and benchmarking betweeen services (Department of Health 2011). Sessional monitoring may promote communciation between patients and therapists to help identify when patients may not be responding to therapy as expected and, consequently, may be more likely to disengage from therapy (Carlier, et al. 2012; Chen et al. 2013; Wolpert et al. 2012). Evidence suggests that sessional monitoring may be associated with higher levels of treatment effectiveness, treatment efficiency, and collaborative practice (Bickman et al. 2011; Gondek et al. 2016; Knaup et al. 2009). Recently, evidence demonstrated a dose–response effect, finding higher levels of treatment effectiveness when feedback was used more often (Bickman et al. 2015). Sessional monitoring can also provide useful information for teams and services to reflect on how their patients are experiencing and responding to therapy (Fleming et al. 2014).

There are a number of barriers to implementing and sustaining sessional monitoring (Boswell et al. 2013; Douglas et al. 2014; Mellor-Clark et al. 2014). Little is known about how it is actually used in routine practice. Sessional monitoring may be less likely with youths with certain demographic and case characteristics. For example, research evidence suggests that measures involving goal formulation at the outset of treatment were more likely to be used with younger youths and youths presenting with emotional difficulties or learning disabilities (Jacob et al. submitted). Therefore, it is important to examine whether demographic and case characteristics are also associated with the use of sessional monitoring.

Published research evidence from qualitative studies suggests that one of the barriers to routine outcome and sessional monitoring may be the view that the measures do not capture the full complexity of issues (Moran et al. 2011; Wolpert et al. 2014). Due to this perception of sessional monitoring, it may be less likely in complex cases, such as those involving a greater number of complexity factors, for instance youths experiencing serious physical health issues, being a victim of abuse or neglect, or living in financial difficulty. In addition, certain complexity factors, such as involvement with social services or youths being under state care, may cause challenges to establishing a therapeutic alliance, which has been suggested as important to facilitate the use of measures in therapy (Stasiak et al. 2012). Hence, sessional monitoring may be less likely in cases where such factors are present.

To the best of our knowledge, there is no existing evidence regarding when sessional monitoring is more likely in CAMHS. Differences in when sessional monitoring is used may have implications for both clinical practice and also the meaningful comparison of services, as more data may be available for certain youths than for others. Therefore, the aim of the present research was to explore whether patient demographic (i.e., age, gender, and ethnicity) and case (i.e., presenting problems and complexity factors) characteristics were associated with the likelihood of using sessional monitoring.

## Method

### Participants and Procedure

As part of the children and young people’s improving access to psychological therapies (CYP IAPT) programme (Wolpert et al. 2011), staff routinely collect demographic, outcome, and experience measures completed by the therapist, youth, and/or carer at assessment, on a session-by-session basis, and at case closure (Law and Wolpert 2014). Data from 12 purposively sampled services were collated as part of an internal audit of the CYP IAPT programme. A favourable ethical approval was received from University College London Research Ethics committee (project ID: 6087/001) and the project was registered with local Trusts.

Overall, the total dataset included *N* = 6801 youths, with data collected from 2011 to 2014. Of these youths, 40 % had attended at least three sessions,^1^ which resulted in a final retained sample of *N* = 2690 youths (level-1) from ten services (level-2). Demographic characteristics are shown in Table 1. There were a number of significant differences between the wider sample of youths attending for fewer than three sessions (*n* = 4111) and the included sample attending for at least three sessions. However, when inspecting the magnitude of these differences, the two samples appear to be broadly comparable as all odds ratios or effect sizes were small; nevertheless, there were more youths attending for more than three sessions from low frequency ethnic groups with a medium odds ratio (Cohen 1988).

**Table 1:** Demographic characteristics

	<3 sessions attended	≥3 sessions attended	Odds ratio or effect size
*N*	4111	2690	–
Female	53 % (2195)***	60 % (1616)	1.31
Age *M(SD)*	12.83 (3.79)***	13.16 (3.71)	0.09
White	61 % (2296)**	58 % (1549)	0.88
Black	5 % (216)***	7 % (192)	1.39
Low frequency groups^a^	8 % (347)***	13 % (353)	1.64
Not stated	26 % (1052)***	22 % (596)	0.83
Mood	61 % (2502)***	53 % (1415)	0.71
Anxiety	48 % (1985)***	43 % (1156)	0.81
Externalizing	41 % (1694)***	26 % (710)	0.51
Self-harm	31 % (1278)***	23 % (630)	0.68
Substance misuse	6 % (256)***	4 % (99)	0.58
Risk to others	15 % (626)***	9 % (242)	0.55
Carer management	21 % (1183)***	21 % (574)	0.67
PTSD	17 % (707)***	13 % (361)	0.75
Eating disorder	13 % (542)**	11 % (288)	0.79
Family relationships	50 % (2045)***	40 % (1073)	0.67
Attachment difficulties	27 % (1125)***	22 % (595)	0.75
Peer relationships	45 % (1845)***	29 % (778)	0.50
Maintaining relationships	15 % (603)***	12 % (311)	0.76
Unexplained physical symptoms	6 % (251)	5 % (143)	0.86
Unexplained developmental difficulties	6 % (247)***	4 % (96)	0.58
Self-care	6 % (256)**	5 % (120)	0.70
Other presenting problems^b^	13 % (538)***	9 % (251)	0.70
Looked after child	6 (247)*	5 % (124)	0.76
Developmental difficulties	8 % (340)*	7 % (184)	0.81
Child in need	10 % (426)***	7 % (187)	0.65
Abuse	17 % (709)***	12 % (327)	0.66
Parental health problem	22 % (899)*	19 % (522)	0.86
Other complexity factors^c^	20 % (831)***	14 % (383)	0.66

Differences between young people <3 sessions attended versus ≥3 sessions attended were compared using independent samples *t* test and *χ* ^2^ tests

*PTSD* post-traumatic stress disorder

* *p* < 0.05, ** *p* < 0.01, *** *p* < 0.001

^a^Ethnic groups occurring with a frequency of <5 % were grouped into “low frequency groups” to avoid including under-powered groups in the main analysis (also see Measures)

^b^Presenting problems occurring with a frequency of <5 % were grouped into “other presenting problems” to avoid including under-powered groups in the main analysis (also see Measures)

^c^Complexity factors occurring with a frequency of <5 % were grouped into “other complexity factors” to avoid including under-powered groups in the main analysis (also see Measures)

## Measures

### Demographic Characteristics

Age, gender, and ethnicity were recorded by services as part of routine data recording. Ethnicity was captured using the categories from the 2001 Census and was generally based on self-report by the parent or the youth. These were grouped for analysis as follows: White (including White British, Irish, and Other White background), Mixed (including Mixed White and Black Caribbean, Mixed White and Black African, Mixed White and Asian, and any other mixed background), Asian (including Indian, Pakistani, Bangladeshi, and Other), Black or Black British (including Caribbean, African, and Other), and other ethnic groups (including Chinese and Other). Ethnicities occurring with a frequency of <5 % were then grouped into “low frequency groups” to avoid including under-powered groups in the main analysis (i.e., Mixed, Asian, and other).

### Case Characteristics

To measure case characteristics, 44 items of the Current View questionnaire (Jones et al. 2013) were used that capture presenting problems and complexity factors. The Current View questionnaire is completed by therapists during an initial assessment appointment, with guidance and training available for scoring. In particular, 30 items capture presenting problems (e.g., “Anxious away from caregivers (Separation anxiety)”). Presenting problems occurring with a frequency of <5 % were grouped into “other presenting problems” to avoid including under-powered groups in the main analysis (i.e., psychosis, elimination problems, mutism, gender discomfort, and adjustment to a physical health problem). In addition, 14 items capture complexity factors (e.g., “Young carer status”). Complexity factors occurring with a frequency of <5 % were grouped into “other complexity factors” (i.e., young carer, learning disability, physical health condition, neurological disorder, child protection plan, refugee or asylum seeker, experience of war, and involvement with youth justice system). Therapists responded to the presenting problem items on a four-point scale from none (0) to severe (3), which were recoded from none to absent (0) and from 1 to 3 to present (1).^2^ Therapists responded to the complexity items as no (0) or yes (1). Number of sessions attended was captured as part of routine data recording (*M* = 12.31, *SD* = 13.58, range 1–151).

### Sessional Monitoring

The number of sessions in which sessional measures were used was captured as part of routine data recording. The use of routine measures in at least two sessions was implemented as part of the CYP IAPT programme (Law and Wolpert 2014). Therefore, sessional monitoring was coded as 1 (any sessional measure used in at least two sessions) or 0 (no sessional measure used in any session). Overall, 49 % (1322) of youths had sessional monitoring and 51 % (1368) did not. Sessional measures included for example, the Revised Children’s Anxiety and Depression Scale (Weiss and Chorpita 2011), the Goal Based Outcomes tool (Law 2011), and the Session Rating Scale (Duncan and Miller 2003). Therapists receive training in selecting and using sessional measures as clinically relevant and appropriate; see Law and Wolpert (2014) for further information.

### Analytic Strategy

To examine the relationship between demographic and case characteristics with sessional monitoring, multilevel logistic regressions were conducted in STATA 12 (StataCorp 2011). Three multilevel logistic regressions were performed predicting sessional monitoring. In Model 1 (the null model), the intraclass correlation coefficient (ICC) was computed to examine the variance explained at the service level. In Model 2, the patient-level demographic characteristics were entered: gender (coded 1 for female); grand mean centred age in line with recommendations (Hox 2010); and White, Black, and low frequency ethnic groups (each dummy coded 1, with not stated as the reference category). In Model 3, the patient-level case characteristics were entered: the 17 presenting problem variables described above (see “Participants and procedure” section) (each dummy coded 1 for present), the six complexity factors, and grand mean centred number of sessions attended to control for the expected relationship between a greater number of sessions attended and a greater likelihood of use of sessional monitoring. The likelihood ratio test was used to compare the fit of subsequent models.

## Results

Results of analyses are shown in Table 2. In Model 1, the ICC revealed that 35 % of the variance in sessional monitoring was explained at the service level with 65 % residual or unexplained variance in sessional monitoring, indicating that multilevel regression was appropriate. Moreover, the amount of service-level variation was relatively large compared to previous research showing therapist effects of 6–9 % in treatment outcome and duration (Lutz et al. 2015).

**Table 2:** Multilevel logistic regression models with demographic and case characteristics predicting sessional monitoring

Parameter estimates	Sessional monitoring												
	Model 1					Model 2				Model 3			
	Estimate (*SE*)		OR	CI		Estimate (*SE*)	OR	CI		Estimate (*SE*)	OR	CI	
Fixed effects													
Intercept	0.15 (0.43)		1.16	0.50–2.68		–0.20 (0.44)	0.82	0.34–1.95		0.09 (0.51)	1.09	0.40–2.98	
Female						0.23 (0.10)*	1.26	1.05–1.53		0.04 (0.11)	1.04	0.84–1.30	
Age						0.09 (0.01)***	1.10	1.07–1.13		0.07 (0.02)***	1.08	1.04–1.12	
White						0.30 (0.11)**	1.35	1.08–1.68		0.32 (0.13)*	1.37	1.07–1.75	
Black						–0.06 (0.23)	0.94	0.60–1.58		0.24 (0.25)	1.28	0.78–2.10	
Low frequency groups						0.15 (0.16)	1.16	0.85–1.59		0.22 (0.18)	1.25	0.88–1.77	
Mood										0.38 (0.13)**	1.46	1.24–2.03	
Anxiety										0.46 (0.13)***	1.59	1.12–1.89	
Externalizing										–0.18 (0.17)	0.83	0.60–1.15	
Self-harm										–0.17 (0.14)	0.85	0.64–1.11	
Substance misuse										–0.19 (0.31)	0.82	0.45–1.50	
Risk to others										0.11 (0.22)	1.12	0.72–1.73	
Carer management										–0.25 (0.18)	0.78	0.56–1.10	
PTSD										–0.01 (0.18)	0.99	0.70–1.40	
Eating disorder										0.08 (0.18)	1.08	0.76–1.53	
Family relationships										–0.13 (0.14)	0.88	0.67–1.16	
Attachment difficulties										0.10 (0.17)	1.10	0.79–1.53	
Peer relationships										0.04 (0.16)	1.04	0.75–1.43	
Maintaining relationships										–0.18 (0.19)	0.83	0.57–1.22	
Unexplained physical symptoms										–0.21 (26)	0.81	0.49–1.35	
Unexplained developmental difficulties										–0.44 (0.31)	0.65	0.35–1.20	
Self-care									0.09 (0.29)		1.10		0.62–1.94
Other presenting problems									–0.27 (0.20)		0.76		0.51–1.13
Child under state care									–1.63 (0.29)***		0.20		0.11–0.34
Developmental difficulties									–0.26 (0.21)		0.77		0.51–1.17
Child in need of social service input									–0.95 (0.24)***		0.39		0.24–0.62
Abuse									–0.17 (0.19)		0.84		0.58–1.23
Parental health problem									0.21 (0.14)		1.23		0.93–1.63
Other complexity factors									–0.30 (0.16)		0.74		0.54–1.01
Number of sessions									0.09 (0.01)***		1.09		1.0–1.10
Variance components													
Service-level variance	1.32 (0.30)					1.33 (0.31)			1.50 (0.34)				

*N* = 2690. *OR* odds ratio, *CI* 95 % confidence interval, *PTSD* post-traumatic stress disorder

* *p* < 0.05, ** *p* < 0.01, *** *p* < 0.001

Adding demographic characteristics in Model 2 significantly improved the model fit compared to the null model but the ICC remained 35 %; likelihood ratio test *χ*^2^(5) = 35.11, *p* < 0.001. In particular, girls were more likely [odds ratio (OR) 1.26] to have sessional monitoring data than boys, youths were more likely (OR 1.10) to have sessional monitoring data with each additional year in age, and White youths were more likely (OR 1.35) to have sessional monitoring data than youths with unstated or missing ethnic identifiers.

Adding case characteristics in Model 3 significantly improved the model fit compared to Model 2 but the ICC increased to 40 %, suggesting that case characteristics explained individual-level, not service-level, variance; likelihood ratio test *χ*^2^(24) = 223.89, *p* < 0.001. In particular, irrespective of other presenting problems, youths presenting with mood or anxiety problems were more likely (OR 1.46 and 1.59, respectively) to have sessional monitoring data than youths presenting without these problems. In contrast, youths under state care or those in need of social service input were less likely to have sessional monitoring data than youths without these complexity factors (OR 0.20 and 0.39, respectively). Finally, in line with expectations, youths attending a greater number of sessions were more likely (OR 1.09) to have sessional monitoring than youths attending fewer sessions.

## Discussion

The aim of the present research was to explore whether patient demographic (i.e., age, gender, and ethnicity) and case characteristics (i.e., presenting problems and complexity factors) were associated with the likelihood of using sessional monitoring in CAMHS. Findings of the present research suggest that there may be differences in the likelihood of sessional monitoring data being available for different groups of youths and families. Although the present research was not able to examine these mechanisms, possible explanations for the findings of the present research are discussed below.

In terms of demographic characteristics, girls were more likely to have sessional monitoring data than boys, which may be in line with evidence that females are more likely to seek mental health treatment (Oliver et al. 2005) and complete questionnaires in general (McCarty 2006) than males. Older youths were more likely to have sessional monitoring data than younger youths. This may reflect the fact that older youths may be more verbal and therefore therapists feel more able to include their views, though the exact mechanism is not clear. The fact that youths with White ethnicities recorded were more likely to have sessional monitoring than youths with unstated or missing ethnic identifiers may reflect the fact that data recording was poorer in general for these youth, and therefore unstated or missing ethnic identifiers were also associated with missing information on sessional monitoring.

In terms of case characteristics, irrespective of other presenting problems, youths presenting with mood or anxiety problems were more likely to have sessional monitoring data than youths presenting without these problems. In contrast, youths under state care or those in need of social service input were less likely to have sessional monitoring data than youths without these complexity factors. These findings are consistent with evidence from qualitative studies suggesting that routine outcome and sessional monitoring may feel more acceptable and relevant to both therapists and service users in cases with more prevalent presenting problems, and be less likely in complex cases (Moran et al. 2011; Wolpert et al. 2014). Similarly, as establishing a therapeutic alliance is an important facilitator to using measures in therapy (Stasiak et al. 2012) perhaps sessional monitoring was less likely when there were challenges to this alliance, such as in cases where there was involvement with social services or youths were under state care. Similarly, therapists may have perceived that available sessional measures were not well suited to monitoring progress for more complex cases. As sessional monitoring may help to identify when patients are not responding to therapy and therefore may be likely to disengage with therapy (Gondek et al. 2016; Kluger and De Nisi 1996), it may be of particular importance to use sessional monitoring with complex cases. Future research should examine whether a wider range of sessional measures is needed targeted to complex cases or whether training would help therapists to select sessional measures in complex cases.

Limitations should be considered when interpreting the findings of the present research. First, we used naturalistic, routinely collected data as opposed to those collected under controlled conditions. Therefore, limitations of confounding variables and selection bias may apply (Gilbody et al. 2002), and future research is needed to replicate the findings presented here, particularly to explore which factors explain the large amount of residual or unexplained variation, such as therapist-level factors. Second, given the inclusion criterion of having attended at least three sessions, there was a relatively small proportion of the wider sample (40 %) included in this study, meaning systematic differences between the two samples may have influenced the present findings (also see Participants and procedure for a discussion of the differences between the wider and included samples). Third, we examined the presence versus absence of at least one sessional measure, and future research should examine whether demographic and case characteristics are associated with the frequency or dosage of sessional monitoring. Finally, the source of non-use of sessional monitoring was not available in the dataset, and future research should interview therapists, youths, and carers when sessional monitoring has not been used to understand reasons for non-use of sessional monitoring.

Notwithstanding the above limitations, the present research is the first to examine when sessional monitoring is more likely in CAMHS. The findings suggest that sessional monitoring is more likely when cases present with more common problems such as mood or anxiety problems but may be less likely when cases present with more complex problems, such as when youths are under state care or in need of social service input. These differences may relate to the likelihood of therapists choosing to use these measures with different populations of services users, or may relate to the likelihood of measures being completed by these different groups. Nevertheless, these findings may have important implications for service comparison, especially in light of the mounting drive to consider the impact and quality of service provision across healthcare through outcome measurement in order to demonstrate transparency and accountability (NHS England 2015). In child mental health, evidence is still needed to inform risk adjustment, or how to adjust for differences in expected treatment outcomes between services with different patient populations. If data quality comparisons in terms of sessional monitoring are considered when comparing services, this may advantage those services who see more youths with less complex difficulties and disadvantage those seeing more complex cases, suggesting that these case characteristics need to be taken into account when considering risk adjustment.

## Footnotes

1 Assuming the first session was assessment and the last session was discharge or case closure, there would be at least one treatment session in which a sessional measure could have been used (Law and Wolpert 2014).

2 If at least one case characteristic item was completed, incomplete items were coded as absent.
